# Evaluation of the effect of small single intravenous dose of amiodarone on the prevention of arrhythmias in patients who underwent coronary artery bypass graft surgery: A randomized controlled trial

**DOI:** 10.1002/joa3.12986

**Published:** 2024-04-05

**Authors:** Simin Azemati, Laleh Dehghanpisheh, Pooya Vatankhah, Saeed Khademi, Mohammad Ghazinour, Sima Eghdami

**Affiliations:** ^1^ Anesthesiology and Critical Care Research Center, Department of Anesthesiology and Critical Care, School of Medicine Shiraz University of Medical Sciences Shiraz Iran; ^2^ Department of Surgery, Section of Cardiac Surgery Shiraz University of Medical Sciences Shiraz Iran

**Keywords:** amiodarone, atrial fibrillation, coronary artery bypass surgery, ventricular fibrillation

## Abstract

**Background:**

Atrial fibrillation (AF) is the most frequent arrhythmia after cardiac surgery causing a range of clinical symptoms and treatments that develop in around one‐third of coronary artery bypass surgery patients. We aimed to evaluate the effect of Amiodarone in preventing arrhythmia in patients undergoing coronary artery bypass surgery.

**Method:**

In this double‐blind randomized clinical trial, 60 patients candidate for coronary artery bypass surgery above the age of 18 were included and randomly divided into two groups of intervention, receiving an infusion of Amiodarone (3 mg/kg) 10 min (in 100 cc Normal saline) before declamping of the aorta, and a control group, receiving 100 cc of saline 10 min before declamping of the aorta. The patient's demographic, clinical features, and hospital and clinical course were recorded.

**Results:**

After undergoing operation, 22 (36.67%) of patients were developed arrhythmia. The Amiodarone group demonstrated significantly lower reperfusion ventricular fibrillation (RVF) rates (26.7% vs. 70%; *p* = .001) and AF occurrence (13.3% vs. 60%; *p* < .001) during the initial 24 h after surgery compared to the placebo group. There was no significant difference between the two groups regarding the need for D/C shock after removing the aortic clamp. (*p* = .117) Furthermore, the intensive care unit stay among the amiodaron group was significantly lower than the control group (2.43 vs. 3.07 days; *p* = .013).

**Conclusion:**

The predictive properties in the administration of single intravenous low‐dose Amiodarone 10 min before the declamping of the aorta can significantly lower the rates of RVF and AF after coronary artery bypass grafting, while also decreasing hospitalization duration.

## Graphical abstract

We demonstrated that the predictive properties in the administration of amiodarone 10 min before the declamping of the aorta can significantly lower the rates of arrhythmia after CABG, while also decreasing hospitalization duration.

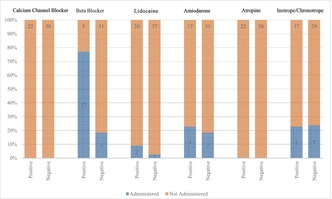

## 1 INTRODUCTION

Coronary artery disease (CAD) is a common cause of mortality worldwide. Coronary artery revascularization is necessary for CAD patients, and in the advanced phases of coronary artery occlusion disease, coronary artery bypass grafting (CABG) is a frequent interventional procedure. CABG is a successful technique, but there might be some challenges both during and after the operation.^1^ The most frequent arrhythmia after cardiac surgery is atrial fibrillation (AF), with a range of clinical symptoms, causes, and treatments that develops in around one‐third of CABG patients.^2^ AF diagnosis is established through clinical indications such as an elevated heart rate, hypotension, an irregular heartbeat, ECG monitoring, and 24‐h Holter monitoring. The best way to treat AF is through a three‐stage assessment process that includes studying the underlying etiology, managing the arrhythmia while lowering the risk of thromboembolism, and finally converting to a normal sinus rhythm.^3^ In the first 5 days following surgery, postoperative AF is most frequently observed,^4^ although it peaks during the first 48 h in cases of open heart surgery, highlighting the necessity for postoperative surveillance.^5^ High risk CAD lengthens stays in intensive care units (ICU) and hospital stays following coronary artery bypass surgery, while also increasing mortality and morbidity.^6^ Age, left ventricular failure, prior episodes of AF, chronic obstructive pulmonary disease (COPD), myocardial infarction (MI), left ventricular size, history of β‐blockers, cross‐clamp duration during surgery, and Cardiopulmonary bypass (CPB) time have all been linked to an increased risk of post CABG AF.^7^, ^8^ Beta‐blockers, amiodarone, anti‐inflammatory medications, statins, and fatty acids are used to treat AF. These treatments prevent AF and manage the arrhythmia while lowering the risk of thromboembolism.^9^

Amiodarone is a distinctive antiarrhythmic medication from class 3 that is used to treat supraventricular, ventricular, and dysrhythmic arrhythmias associated with acute myocardial infarction. Intravenous or oral dosing of Amiodarone for 3–5 days before surgery and after coronary artery bypass surgery can decrease AF incidence.^4^ However, studies have reported various results following different doses and routes of administration, along with the timing of initiation in relation to cardiac surgery (e.g., pre‐, peri, or postoperative).^4^

In this study, we evaluated the efficacy of a single low‐dose of Amiodarone in preventing AF following coronary artery bypass surgery based on its antiarrhythmic properties in lowering the incidence of AF.

## 2 MATERIALS AND METHODS

### 2.1 The study design

The present study is a double‐blinded randomized controlled trial among coronary artery bypass graft patients. This multicentral study location was Hospitals affiliated with the Shiraz University of Medical Science, Shiraz, Iran.

The inclusion criteria consisted of patients above 18 years of age, who completed written consent to participate in the study and were candidates for CABG under CPB. Patients were excluded in cases of diabetes, hypothyroidism or hyperthyroidism, liver failure, abnormal laboratory data such as creatinine of above 2, increased SGOT or SGPT over twice, patients with chronic obstructive pulmonary diseases, preoperative AF, pregnancy, Redo or combined surgery, and EF lower than 40%.

### 2.2 Surgical intervention

All patients underwent similar induction method, in which sodium thiopental 3 mg/kg, Pancuronium bromide (0.2 mg/kg), Sufentanil (0.5 μg/kg), morphine (0.1 mg/kg), and midazolam (0.1–0.2 mg/kg) was administered. Propofol with a dose of 100 μg/kg/min was used to maintain anesthesia. The administered antibiotics during anesthesia were meropenem (1 g) and clindamycin (600 mg). Anticoagulants included heparin with a dose of 300–400 U/kg before cardiopulmonary bypass. The activated clotting time (ACT) was checked after 5 min and an ACT of above 480 was used as the cut‐off for initiation of cardiopulmonary bypass.

The prime solution was similar for all patients in the control and intervention groups, which includes ringer lactate solution and modified gelatin, along with 100 U/kg heparin. The prime was heated to a temperature of 35°C. Cardioplegia was repeated every 20 min by antegrade method and consisted of a combination of hypothermic blood and crystalloid.

The surgical technique was similar in all patients and was performed by a same team in all cases, grafts were obtained from the left internal mammary artery (LIMA) and the saphenous vein. At the end, after patient separation from the pump, to inverse heparin effect, protamine sulfate was given at the rate of 50 mg per 5000 units of heparin, and ACT was checked accordingly.

### 2.3 Intervention and allocation

In this double‐blind study, 60 patients who met the criteria for entering the project were selected. Patients were randomly divided into two groups. In the intervention group, Amiodarone (HAMELN‐GERMANY) (3 mg/kg) diluted in 100 cc of normal saline (N.S) was added to the pump tank and infused 10 min before decamping of the aorta, while in the control group patients received 100 cc of N.S in their pump tank 10 min before decamping of the aorta (Placebo group). Within 30 min after removing the aortic clamp, the first cardiac rhythm until sinus rhythm appeared was recorded. In the case of ventricular fibrillation (VF), D/C shock was used, while if an atrioventricular block occurred, a pacemaker was placed. We aimed to evaluate the effect of Amiodarone in preventing arrhythmia in patients undergoing coronary artery bypass surgery.

### 2.4 Data collection

Any occurrence of arrhythmia was documented during the first 24 h after CABG surgery while the patient was admitted to the intensive care unit (ICU). Patients recorded data included their demographic features, along with surgical features, clinical and laboratory data such as fasting blood sugar (FBS) and blood sugar (BS), blood urea nitrogen (BUN), creatinine (Cr), aspartate aminotransferase (AST), alanine transaminase (ALT), calcium (Ca), magnesium (Mg), sodium (Na), and potassium (K).

### 2.5 Statistical analyses

The sample size was calculated by considering a type I error of 5% and a power of 80%, and by considering the risk of drop‐out, a total of 60 patients (30 in each group) was considered for our study. Data were entered into SPSS version 26.0 and evaluated regarding normal distribution via the Kolmogorov–Smirnov test. Data are presented as frequency and percentage (%) or mean and standard deviation (SD). The independent sample *t*‐test was used for the analysis of continuous variables while the Chi‐square/Fisher's exact test was used for categorical variables. A *p*‐value of less than .05 was considered statistically significant.

## 3 RESULTS

In this study, 60 patients candidate for CABG surgery were included and randomly divided into two groups, the placebo and Amiodarone groups. Table 1 demonstrates the baseline data of the groups in our study. As demonstrated, there was no statistically significant difference among the two groups regarding the age, gender, weight, BSA, EF, preoperative laboratory data (hematocrit, K, BUN, Creatinin), comorbid disease (HTN, MI), and drug history (beta blocker, ACE inhibitor) (*p* > .05). Also, there was no statistically significant difference regarding surgical features such as the number of vein grafts, use of LIMA, inotrope, K levels at the baseline and after the removal of aortic cross‐clamp, CPB time, and the duration of surgery (from the incision of the chest wall till its closure). (Table 1) The patients were also similar regarding hemodynamic features after anesthesia induction and also after weaning from CPB among the two groups.

**TABLE 1:** Comparison of demographical and anesthesia features based on intraoperative administration of Amiodarone or placebo among patients undergoing cardiopulmonary bypass surgery.

Variable		Group		*p*‐value
		Amiodarone; *n* = 30	Placebo; *n* = 30	
Baseline features				
Age (years); mean ± SD		58.5 ± 9.5	59.8 ± 10.1	.609
Sex; *n* (%)	Male	26 (86.7)	21 (70.0)	.117
	Female	4 (13.3)	9 (30.3)	
Weight (kg); mean ± SD		70.8 ± 11.8	70.8 ± 10.7	.991
Height (cm); mean ± SD		167.9 ± 8.0	163.6 ± 6.6	**.027** *
BSA (m^2^); mean ± SD		1.8 ± 9.2	1.7 ± 9.1	.313
Hypertension; *n* (%)		10 (33.3)	15 (50.0)	.190
Myocardial infarction; *n* (%)		12 (40.0)	13 (45.0)	.309
ACE inhibitor; *n* (%)		33.3%	43.3$	.426
Beta‐blocker; *n* (%)		9 (40.0)	10 (45.0)	.436
Hematocrit (%); mean ± SD		38.39 ± 4.5	37.0 ± 4.4	.238
BUN (mg/dL); mean ± SD		16.2 ± 4.0	14.4 ± 3.1	.061
Creatinine (mg/dL); mean ± SD		1.1 ± 0.2	1.1 ± 0.2	.513
Aspartate transaminase (u/L); mean ± SD		22.43 ± 6.42	23.23 ± 6.75	.650
Alanine transaminase (u/L); mean ± SD		23.63 ± 7.29	22.9 ± 6.30	.678
Operation features				
Potassium (mmol/L)	Baseline	4.26 ± 0.50	4.33 ± 0.42	.561
	At the release of aortic cross‐clamping	4.59 ± 0.44	4.80 ± 0.52	.100
Cross‐clamp (min); mean ± SD		32.57 ± 10.25	33.37 ± 7.34	.680
CPB time (min)		54.71 ± 13.4	56.93 ± 11.77	.390
Surgery time (min)		155.3 ± 20.28	156.65 ± 18.29	.630
Inotrope; *n* (%)		23 (76.7)	24 (80.0)	.754
LIMA; *n* (%)		3 (10)	2 (6.7)	.640
Vein graft; mean ± SD		85.6 ± 17.4	92.5 ± 12.7	.086
Hemodynamic features				
After anesthesia induction	Heart rate	81.1 ± 13.31	79.9 ± 10.81	.703
	MAP	77.8 ± 13.81	76.00 ± 14.32	.622
	CVP	8.70 ± 3.03	8.80 ± 3.86	.882
After weaning from the cardiopulmonary pump	Heart rate	85.6 ± 17.37	92.5 ± 12.71	.086
	MAP	66.8 ± 9.67	72.4 ± 11.8	.051
	CVP	7.2 ± 3.14	8.4 ± 3.08	.120

Abbreviations: BSA, Body surface area; BUN, blood urea nitrogen; CPB, Cardiopulmonary bypass; LIMA, Left internal mammary artery; SD, Standard deviation.

* Chi‐square/Fisher's exact test or independent sample *t*‐test.

Among the patients in our study, 22 (36.67%) developed AF after the operation. The patients were evaluated regarding cardiac complications. (Table 2) As demonstrated, there was a significant difference in the occurrence of AF in the ICU during the initial 24 h after operation among the two groups, in which the rate was significantly lower in the Amiodarone group (13.3% vs. 60%; *p* < .001). Furthermore, the Amiodarone group demonstrated significantly lower RVF rates compared to the control group. (26.7% vs. 70%; *p* = .001) There was no significant difference between the two groups regarding the need for D/C shock after the removal of the aortic clamp. (*p* = .117) Furthermore, the ICU stay among the Amiodarone group was significantly lower compared to the control group (2.43 vs. 3.07 days; *p* = .013).

**TABLE 2:** Comparison of post‐operative features based on intraoperative administration of Amiodarone or placebo among patients undergoing cardiopulmonary bypass surgery.

Variable		Group		*p*‐value
		Amiodarone; *n* = 30	Placebo; *n* = 30	
Postoperative features				
Atrial fibrillation; *n* (%)		4 (13.3)	18 (60.0)	**<.001** *
RVF; *n* (%)		8 (26.7)	21 (70.0)	**.001** *
D/C Shock; *n* (%)		4 (13.3)	9 (30.0)	.117
ICU stay (days); mean ± SD		2.43 ± 0.77	3.07 ± 1.11	**.013** *
Hospital stay (days); mean ± SD		4.23 ± 0.94	4.83 ± 1.76	.105
FBS (mmol/L); mean ± SD		100.63 ± 13.29	106.37 ± 11.09	.112
BS (mmol/L); mean ± SD		127.83 ± 34.27	124.97 ± 22.13	.734
Aspartate transaminase (u/L); mean ± SD	Baseline	22.43 ± 6.42	23.23 ± 6.75	.650
	After Operation	29.47 ± 8.76	30.63 ± 11.01	.625
Alanine transaminase (u/L); mean ± SD	Baseline	23.63 ± 7.29	22.9 ± 6.30	.678
	After Operation	22.87 ± 8.58	24.17 ± 9.08	.612

Abbreviations: BS, Blood sugar; FBS, fasting blood sugar; ICU, intensive care unit; LIMA, Left internal mammary artery; SD, Standard deviation; RVF, reperfusion ventricular fibrillation.

* Chi‐square/Fisher's exact test or independent sample *t*‐test.

**FIGURE 1:**
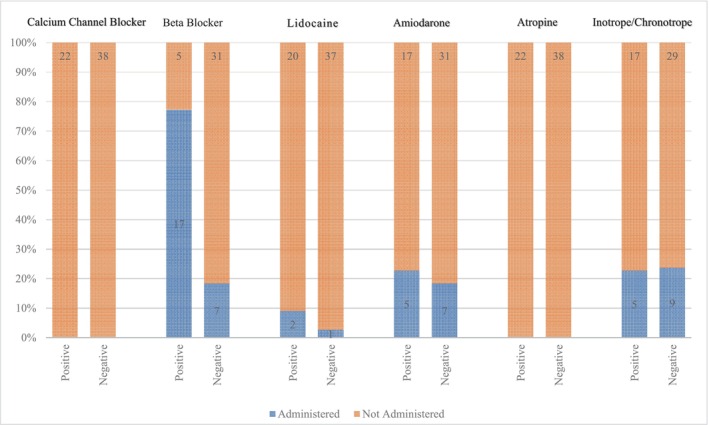
Evaluation of administered drugs in the intensive care unit based on the development of arrhythmia.

We also evaluated the use of inotropes and anti‐arrhythmic drugs in the ICU. In this regard, calcium blockers, beta‐blockers, lidocaine, Amiodarone, and atropine drug usage and frequency were evaluated based on the occurrence of arrythmia (Figure 1). Based on our results, only beta‐blockers were significant associated with developing cardiac arrhythmia (*p* < .001). In other words, beta‐blockers were administered in 17 (77.3%) out of the 22 patients who developed an arrhythmia.

## 4 DISCUSSION

Previous studies have been conducted on the effect of Amiodarone on arrhythmia, however, they mainly focused on preventing the occurrence of arrhythmia in patients undergoing open heart surgery in the form of oral dose or intravenous dose, or intravenous infusion before, during, and after surgery. Here we evaluated the efficacy of a single low dose of Amiodarone in preventing AF following coronary artery bypass surgery. in order to achieve lowering the incidence of AF during the first 24 h in the ICU. Induction settings and parameters were similar for both groups in our study. The incidence of RVF within 30 min after removing the aortic clamp was significantly lower in the Amiodarone group compared to the placebo group (26.7% vs. 70%; *p* = .001). The study found that low‐dose administration of Amiodarone had a similar effect to a report by Samantaray et al.^10^ regarding the use of low‐dose Amiodarone (150 mg) 3 min before the removal of the aortic clamp from the pump while evaluating the incidence of RVF within the first 30 min. However, in a report by Buckley et al.,^4^ although not significant, low‐dose administration of Amiodarone had higher odds of developing AF compared to the medium and high dose groups. Various adverse effects following Amiodarone administration should be taken into consideration and reduce the long‐term side effects.^11^ In a study by Kochiadakis et al.^11^ regarding post‐operative administration of Amiodarone with a dose of 200 mg/day, 15 patients experienced significant adverse effects, including symptomatic bradycardia hypothyroidism, hyperthyroidism, and ataxia, while 9% of patients developed minor side effects such as sun sensitivity, gastrointestinal discomfort, nausea, and ophthalmic problems. Patients receiving high doses of Amiodarone have reported more side effects like bradycardia and excessive QT prolongation.^12^, ^13^, ^14^ Also, due to the economic and public constraints on timing of surgery, delaying patients’ operations to achieve a satisfactory preoperative level and loading regimen of Amiodarone is impractical, and the perioperative period, such as the case in our study, may be to most suitable phase for the administration of the drug. Therefore, proper patient selection along with adjusting the most optimal route and dosage is essential to obtain the utmost benefits in the management of cardiac surgeries.

Patients with AF and rheumatic heart disease were found to benefit from low‐dose oral Amiodarone in terms of restoring and maintaining normal sinus rhythm following balloon mitral valvotomy.^15^ The incidence of AF and the need for cardioversion, defibrillation, and energy was significantly lower following single intraoperative dose administration of Amiodarone in patients undergoing cardiac valve replacement surgery.^15^, ^16^ Amiodarone significantly reduced the ventricular rate even in patients whose rhythm was not converted to normal sinus rhythm.^17^ Amiodarone may therefore be useful in either changing the rhythm to normal sinus rhythm or regulating the fast ventricular rate. This therapy can frequently keep patients with left atrium dimensions between 46 mm and 60 mm, who are significantly compromised by AF in normal sinus rhythm.^18^

Another post‐operative factor that was evaluated in our study was the frequency of D/C Shock. Samantaray et al.^10^ reported a higher rate of D/C shock in the Amiodarone group compared to the placebo group (65% vs. 18%), although not statistically significant. These findings were contrary to our results demonstrating higher rates of D/C shock in the placebo group compared to the Amiodarone group (30% vs. 13%). Although we did not reach a statistical significance in this regard, its clinical importance cannot be overlooked.

One of the factors which should be taken into consideration during Amiodarone administration is its effect on liver enzymes, particularly AST and ALT. Studies have highlighted the significance of monitoring a patient's liver function tests and mental state during IV administration of Amiodarone.^19^ *N*‐acetylcysteine treatment has been reported in the treatment of acute liver failure in these cases.^20^ However, in our study and based on our pre‐and post‐operative data, no significant increase was observed in these liver function tests in the Amiodarone group compared to the placebo group.

Another finding in our study was the preventive effect of Amiodarone on the occurrence of AF during the initial 24 h after surgery. Based on our results, Amiodarone demonstrated significantly lower rates of AF compared to the control group (13.3% vs. 60%; *p* < .001). These findings are in line with other studies.^6^, ^10^, ^21^ In a related study conducted by Kojuri et al. (2009) on 240 patients underwent CABG from Iran, they found that both propranolol and Amiodarone were effective in lowering the incidence of AF following CABG, but that Amiodarone or the combination of Amiodarone and propranolol as a prophylactic drug was more effective than propranolol alone.^22^

The study found that the average stay in the ICU of patients in the Amiodarone group was significantly lower than the placebo group. This result is consistent with the research of Budeus et al.^6^ but contradicts the findings of Treggiari‐Venzi et al.^21^ However, regarding hospital stay, although the patients in the amiodarone group had a shorter period of hospital stay compared to the placebo group, this difference was not statistically significant. Nonetheless, the study of Treggiari‐Venzi et al.^21^ is in line with the findings of this study in this regard. Beta‐blockers were the most administered medication among patients in the ICU who developed AF (17 out of 22; *p* < .001). These results are inconsistent with the findings of Kamali et al.,^23^ which examined the preventive effects of Amiodarone and metoprolol on lowering the occurrence of AF after CABG. According to the study's findings, metoprolol greatly outperformed Amiodarone in reducing the incidence of AF during CABG. In a different study, Lamb et al.^24^ supported atenolol's role in the prevention of supraventricular arrhythmias after CABG, especially for patients with adequate left ventricle function. In a different study by Halonen et al.,^25^ AF was seen in 23.9% of metoprolol‐treated patients and 28% of Amiodarone‐treated patients. (*p*‐value = .85). Another study by Johnson and Brophy^26^ found that those who took sotalol had fewer instances of AF and other related disorders. Another study by Esmail et al.^27^ showed that administering Amiodarone even in doses lower than those recommended in different sources can prevent the development of post‐CABG AF. Due to limited evidence in this regard, further studies regarding the preventive role of beta‐blockers in post‐CABG patients are justified.

Among the limitations of our study are the limited sample size and the single‐center nature of our study. Therefore, further multicentral and larger populational studies, along with meta‐analytic studies are needed to reach an ultimate assumption.

## 5 CONCLUSION

We demonstrated the predictive properties in the administration of a single intravenous low dose Amiodarone 10 min before declamping of the aorta can significantly lower the rates of arrhythmia after CABG, while also decreasing duration of hospital stay. Further meta‐analytic studies are needed to determine the exact application and preventive roles of Amiodarone, and also beta‐blockers, in CABG patients.

## AUTHOR CONTRIBUTIONS

P.V. and L.D. designed the study. S.A. collected the data. S.K. performed a review of the literature and drafted the manuscript. All authors proofread and accepted the final version of the manuscript.

## FUNDING INFORMATION

No financial fund was received for this report.

## CONFLICT OF INTEREST STATEMENT

The authors declare that they have no competing interests.

## ETHICAL APPROVAL

This study was approved by the ethics committee of Shiraz University of Medical Sciences (Code: IR.SUMS.Med.RED.1394.205) and also approved by the registry for clinical trials (Code: IRCT2016053014372N7; registered at 29/6/2016, available at: https://en.irct.ir/trial/14039?revision=14039). The study was conducted in compliance in accordance with the relevant guidelines and regulations and the Declaration of Helsinki. Written informed consent is obtained from all patients to participate in this study after giving complete information about the disease, existing treatments and current treatment, and its possible effects and complications. Patients are free to leave the study at any time. Also, the confidentiality of the patient's information was assured by the researcher.

## CONSENT FOR PUBLICATION

Not applicable.

## CLINICAL TRIAL REGISTRATION

This study was approved by the ethics committee of Shiraz University of Medical Sciences (Code: IR.SUMS.Med.RED.1394.205) and also approved by the registry for clinical trials (Code: IRCT2016053014372N7; registered at 29/6/2016, available at: https://en.irct.ir/trial/14039?revision=14039).

## Data Availability

The findings of the present study are available on request from the corresponding author. They are not publicly available due to privacy and ethical restrictions.
